# Is it possible to control bovine tuberculosis without compensation? Reviewing ten years of the Chilean program and its progress

**DOI:** 10.1186/s13620-023-00243-y

**Published:** 2023-08-24

**Authors:** Nicolás Valdivieso, Patricio Retamal

**Affiliations:** 1 Servicio Agrícola Y Ganadero, Ministerio de Agricultura, 8330246 Santiago, Chile; 2 Facultad de Ciencias Veterinarias Y Pecuarias, Universidad de Chile, 8820808 Santiago, Chile

**Keywords:** Bovine tuberculosis, Livestock, Official program, Agricultural service, Surveillance

## Abstract

In 2011, the Chilean bovine tuberculosis (bTB) program was launched by the Livestock and Agriculture Service (SAG) as a compulsory countrywide program based on testing and culling of bTB reactors at herd-owners expense. This review outlines the rationale and key components of the bTB program, and the dynamic changes that have occurred since 2011. The paper also examines the problems identified by stakeholders and the initiatives put in place to address the constraints to achieving progress.

To date, the program has shown progress in controlling bTB. However, in order to achieve bTB eradication it will be essential to improve the commitment of stakeholders, and to develop a framework of strong and workable regulations that will help to manage bTB outbreaks, particularly where clusters of bTB infection are recorded.

## Background

Bovine tuberculosis (bTB) is a worldwide-distributed disease with incidence levels that vary depending on a range of geographical and environmental factors, as well as production systems and a host of animal and herd risk factors [1]. In Chile, there have been reports of bTB since 1893, providing an early warning call for the need to initiate control and prevention strategies [2]. However, it took until 2011 for stakeholders to reach an agreement when the national program for the control and eradication of bovine tuberculosis was launched. The impacts on economic losses, barriers to exports and the country's image were the key drivers for the national program, which was based on testing and eliminating bTB reactor animals at the producers’ expense [2–7].

**Fig. 1:**
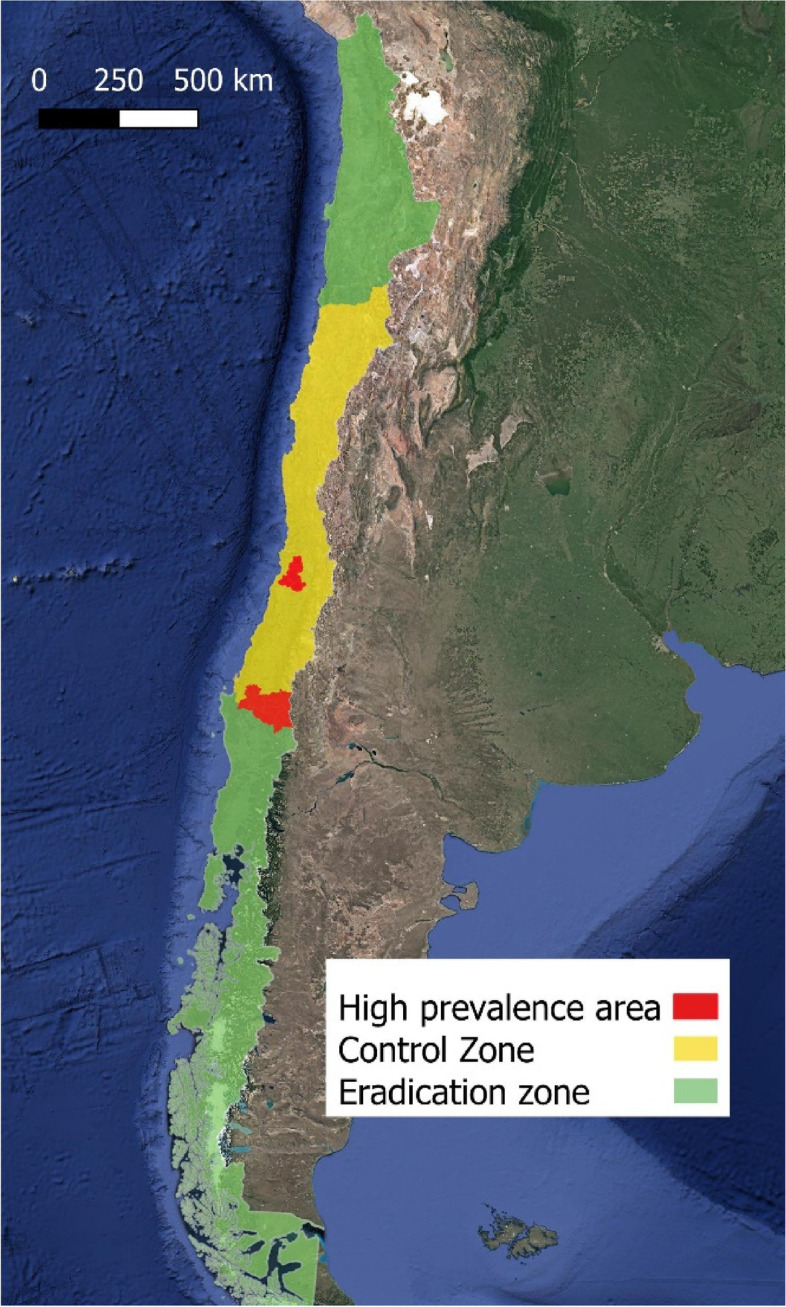
A map of Chile highlighting the bTB program zones, including the control zone (centrally, with the high prevalence areas) and the eradication zones (northern and southern)

In order to deal with differences in prevalence and production systems throughout the country, regional zoning was applied to provide specific and effective strategies to control and eradicate bTB [7]. The ‘eradication zone’ was established in the south of the country, where the majority of dairy farming systems are located, and where the prevalence of infected farms (4.7%) is the lowest in the country. Meanwhile, the region of central Chile was designated as the control zone, which clustered up to 85.5% of infected farms (Fig. 1).

According to the latest livestock census in 2021, there are 125,408 bovine herd owners in Chile and a total population of 3,718,532 cattle, with 71% (2,672,493) of the cattle population located in the southern eradication zone [4]. Because of the nature of available grazing areas, the cattle population in Chile is distributed among a small proportion of herd owners, with 7% of all ranchers farming 60% of the cattle population. The remaining 40% are farmed by small and medium-sized ranchers [5].

Due to the high costs of the testing and culling borne by producers in the control area (where tuberculosis is more prevalent and the systems are oriented to calf rearing and fattening), the implementation of the program has experienced difficulties in maintaining surveillance measures and cleaning up of infected herds [1]. Therefore, it is necessary to review the results of the national control program, by comparing the results between the different epidemiological zones after 10 years of a program that, importantly, does not include compensation for culling of reactor animals.

To carry out this review, the official data was extracted from the Livestock and Agriculture Service (SAG) annual reports of animal diseases surveillance and directly from the Animal Health System (SSA), the web platform on which veterinarians (official and private) record laboratory and skin test results. The objective of this review is to identify and clarify the various components of the program and evaluate the surveillance data in order to understand the successes and constraints of the Chilean bTB program.

## Chilean TB program brief overview

Official inspection teams work in the country's 62 slaughterhouses, inspecting carcasses and verifying their suitability for human consumption. All visible lesions (VL), compatible with bTB are sent to the official laboratory for PCR, culture, and histopathology. This is the principal way in which the largest number of infected herds have been detected. Elsewhere, official and accredited veterinarians carry out the tuberculin skin test in the field, where all herds with reactor animals are investigated, and skin test positive animals are sent to the slaughterhouse for post-mortem inspection for lesions. The skin tests are carried out as part of the surveillance program within dairy farms, as well as providing certification for export from bTB free farms.

During the 1980s, the public and private sectors managed the first action points for bTB control; the development of a bTB—free herd certification scheme; a voluntary binding agreement with dairy farms where SAG certifies that bTB is absent, and the milk industry pays a bonus price per liter to the producer. This is a key part of the program since it is the only major incentive for dairy producers to screen and manage the bTB infection risk, and also because this certification process covers approximately 70% of the tuberculin skin tests that are performed in the field.

In 2011 the bTB program was launched with a package of compulsory rules to notify, control and screen herds for bTB, depending on the epidemiological risk zone. The regional zoning took into account the regional disease incidence and bovine population data by using a clustering model to support the decision-making processes of the two epidemiological zones as part of the National Tuberculosis Management Plan (Fig. 1) [8]. This required that specific regulations were put in place to control animal movement, a clean-up bTB scheme, herd surveillance, all with the aims to control and eradicate the disease in the respective epidemiological zones.

In a cluster and risk analysis carried out by Rivera et al., in 2015 [5], two areas with a very high risk of exposure and associated high bTB prevalence were identified within the control zone. Despite the state's interest in updating and monitoring the regulation of cattle movements based on risk, as well as incorporating more control and oversight of breeding systems, only a limited number of modifications have been made to the rules that establish and regulate surveillance activities in the control zone.

The Official Traceability program started in Chile in 2005; it was set up as a public–private initiative and instigated as a requirement to facilitate export meat products and live animals. Over time, this major program for all disease surveillance and control actions has been strengthened with a coding system for identification of premises, animal identification through use of Radio Frequency Identification tags and recording of animal movement, among other compulsory regulations for bovine herd owners.

Due to the absence of a financial compensation policy for infected or reactor animals, it was realized that new tools were required to deal with highly infected herds. Therefore, in 2016, the SAG in partnership with the University of Chile, conducted research projects to assess the efficacy of the *Mycobacterium bovis* BCG vaccination of cattle, which has since evolved into a pilot bTB cattle vaccination strategic plan targeting high-infected dairy herds within the control zone.

## Findings

**Fig. 2:**
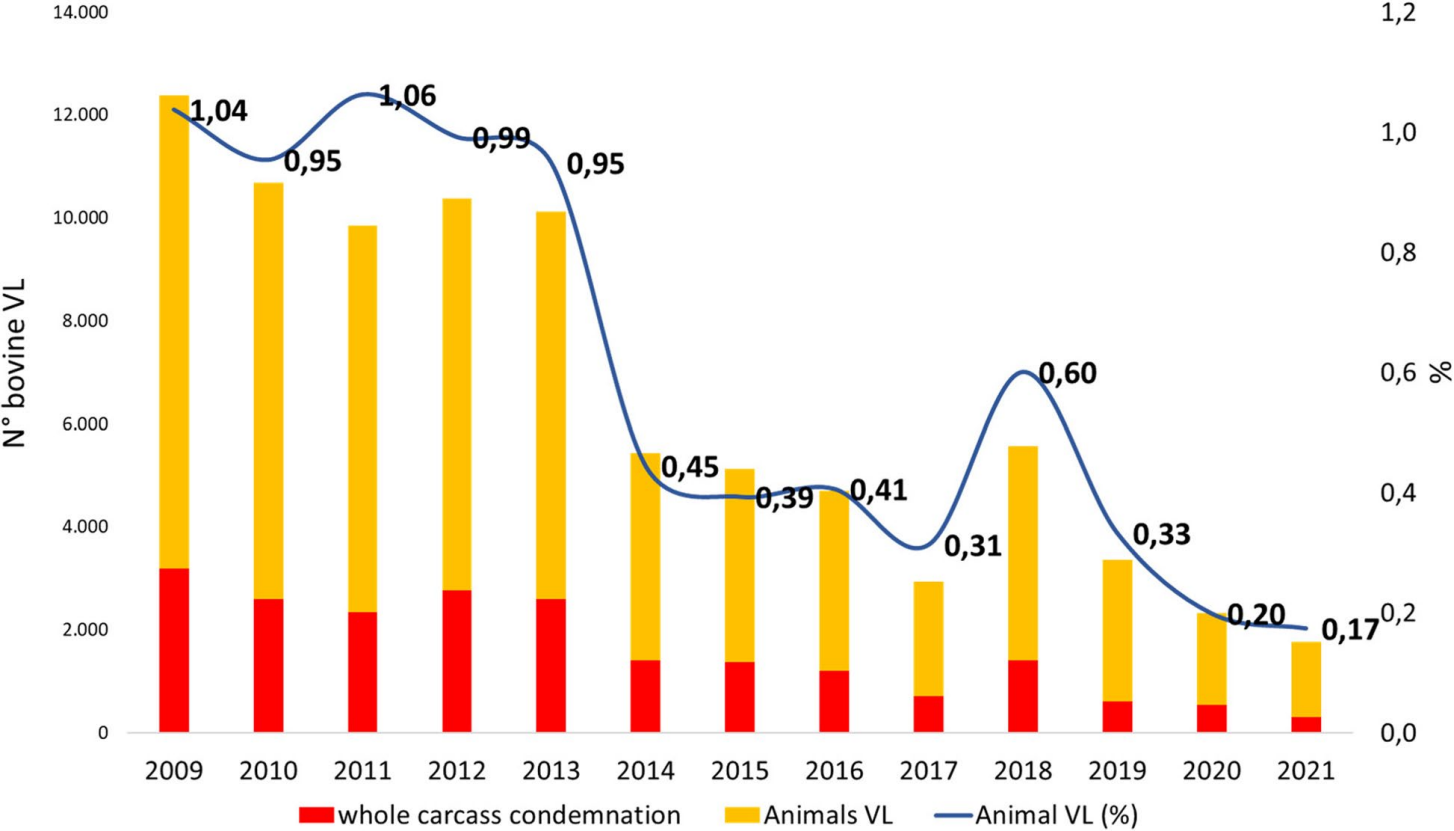
The number and percentage of slaughtered animals each year that were deemed bTB-infected following visual lesion (VL) detection. Following VL detection, condemnation of carcasses was either partial (yellow) or whole (red)

In Chile, since the 1970s the routine inspection of carcasses has been carried out at slaughterhouses [3] where, depending on the severity and distribution of visible lesions (VL), either total or partial carcass condemnation occurs where VL consistent with bTB are disclosed [6]. The annual national carcass condemnation rate has shown a downward trend of bTB compatible VL, ranging from from 1.04% to 0.17% of the total bovines slaughtered per year [Fig. 2].

From analysis of this data it has also been possible to observe the increase in the VL and condemnation rate that occurred in 2018; this was likely due to the largest dairy in the country experiencing a disease control failure arising from the management of the milk pasteurizer. This resulted in the transmission of bTB infection to the replacement heifers, with consequent increased condemnation rates that impacted on the national bTB figures.

**Fig. 3:**
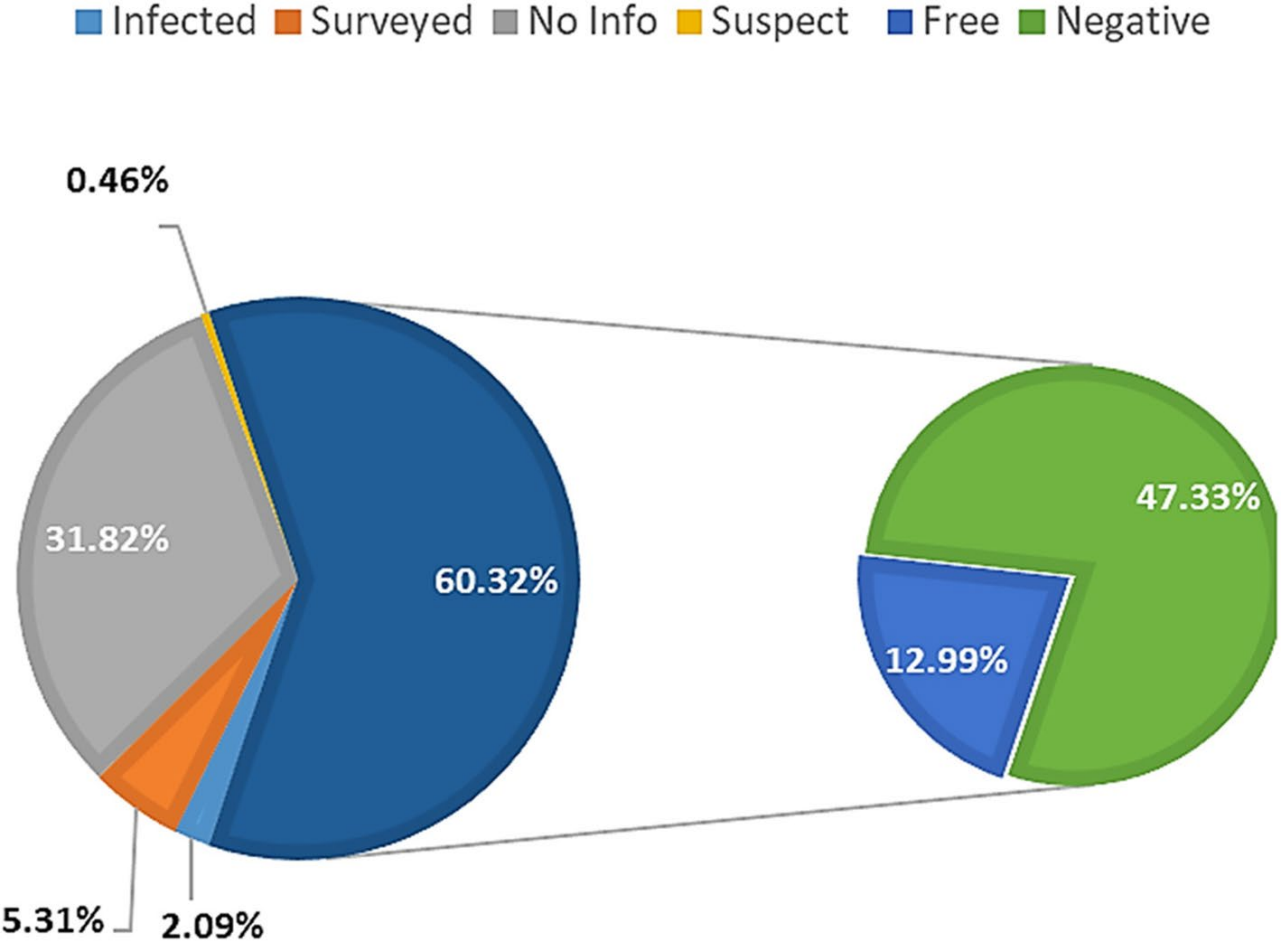
The distribution of cattle farms by bTB classification, for all farms within the national surveillance program

The distribution of bTB classifications at farm level is presented in Fig. 3. There are 5 classes incorporating a total of 19,297 cattle farms with surveillance records [9]:

- *Negative*: 11,639 (60.3%), where there has been active surveillance with negative results (negative non-certified, 47.3%; certified bTB free, 13%).
- *Surveyed*: 1,025 (5.3%), those that have been surveyed (2011–2021) either at *post- mortem* inspection or by checks in the field. Although surveyed farms have negative bTB results, the information is not sufficient for them to be classified as negative farms.
- *Suspect*: 89 (0.5%) farms under investigation due to positive bTB surveillance in the field or at *post-mortem*.
- *Infected*: 403 (2.1%), farms where bTB infection has been confirmed. It is notable that 65 (16.1%) of these infected farms are dairy enterprises, and 338 (83.9%) are breeding and/or fattening farms.
- *No information*: 6,141 (31.8%) i.e., those farms that haven’t had bTB surveillance records, but may have other disease surveillance records, for example, brucellosis infection data.

If all the farms in the livestock census were included in this classification, from the total number of farms that own cattle, 10.5% have no record of bovine tuberculosis surveillance (Table 1).

**Fig. 4:**
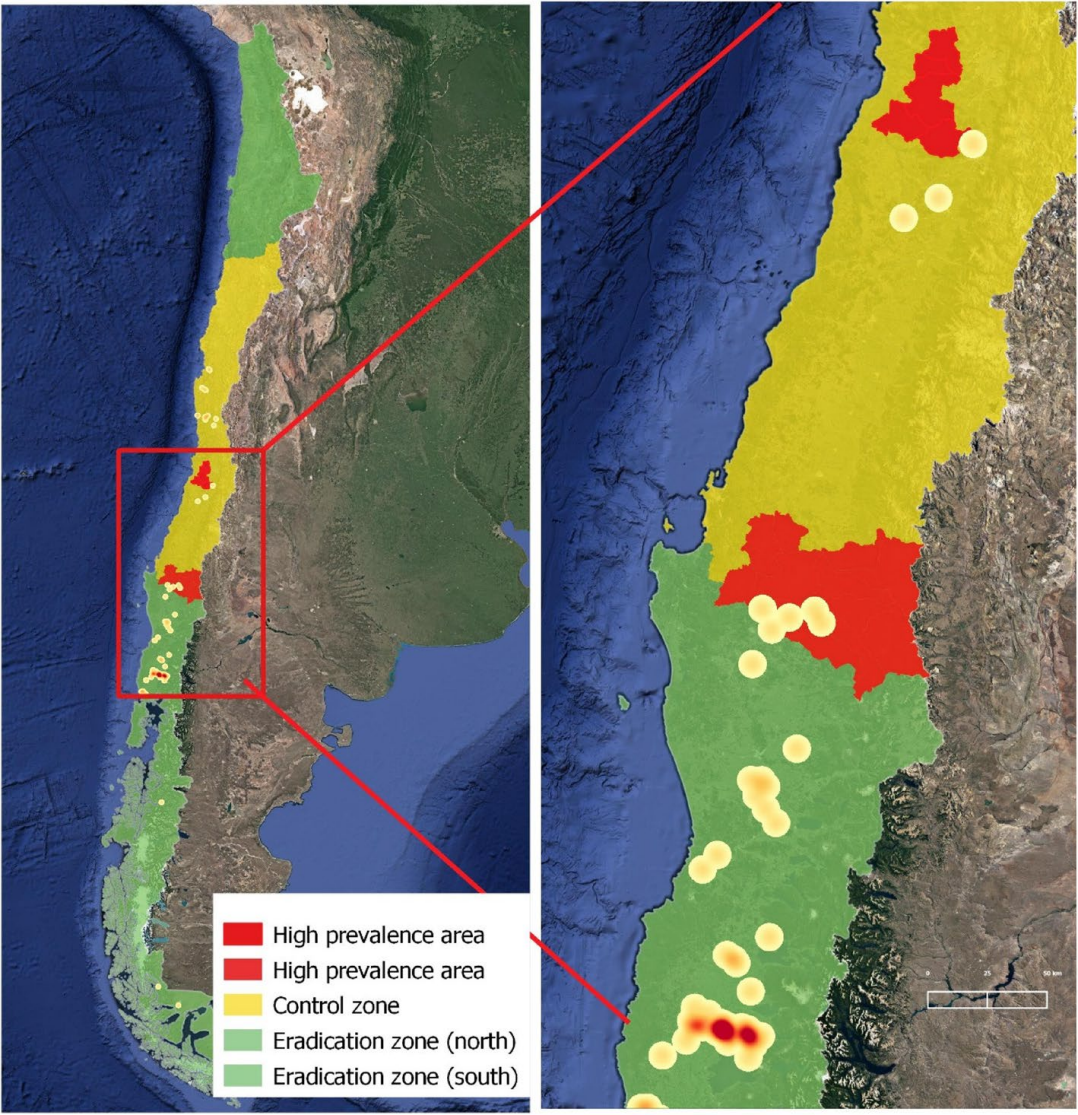
Heat map of bTB infected herds in Chile during 2011

**Fig. 5:**
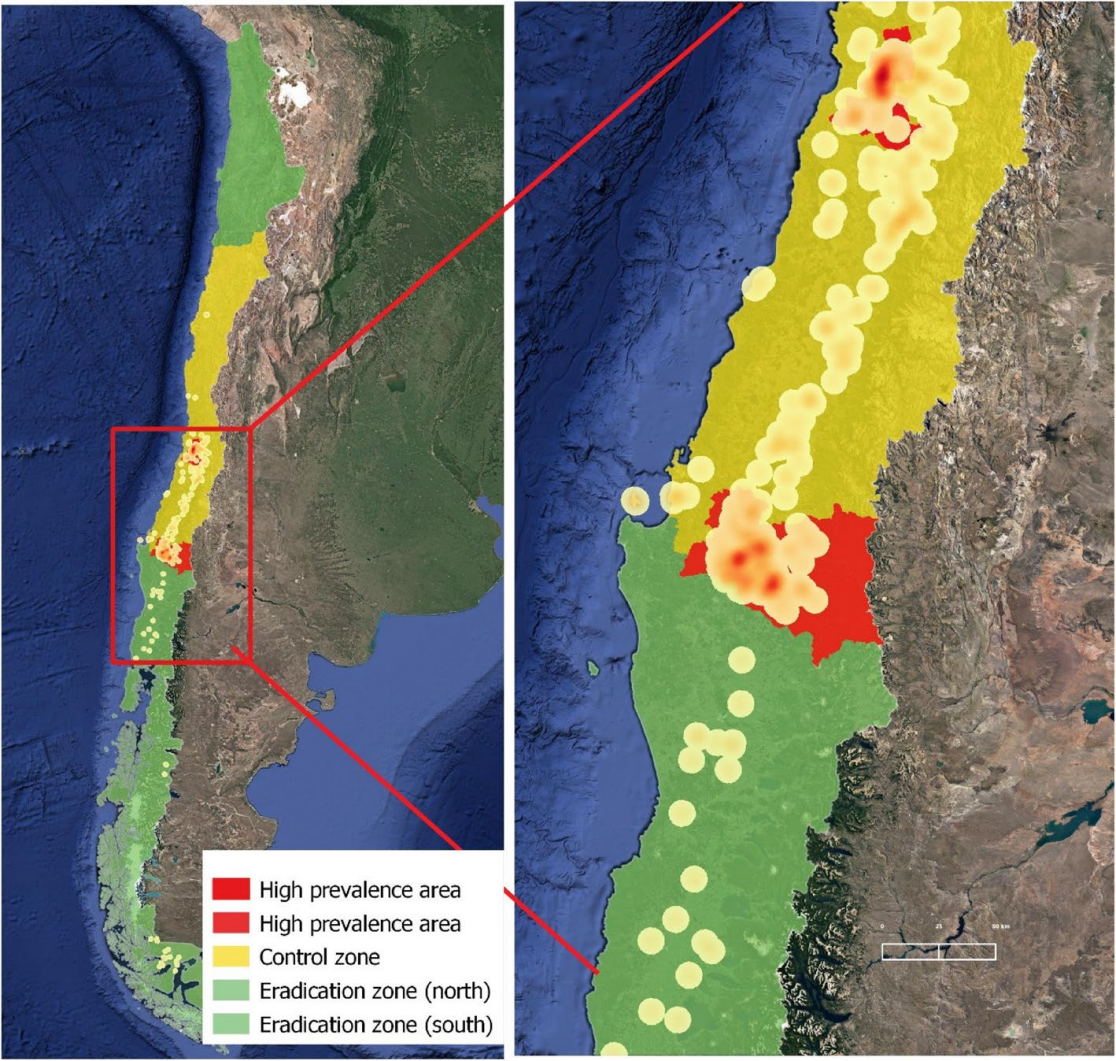
Heat map of bTB infected herds in Chile during 2021

The bTB heat map in Fig. 4 shows that, in 2011, the infected herds were concentrated in the majority dairy farm areas in Los Lagos and Los Ríos Region, in the central-south of the country. However, there is limited comparable information on confirmed bTB infected herds in the central area. After the first 10 years of the National Plan, the bTB levels at farm level have diverged when comparing the control and eradication zones, with prevalence levels of 1.4% and 0.08%, respectively. In the heat map for 2021 (Fig. 5), there were still two infection clusters identified in the control zone, corresponding to those areas defined with high bTB prevalence. In the southern eradication zone, there were also infected farms, but no evidence of clustering that indicated specific risk factors for infection.

**Fig. 6:**
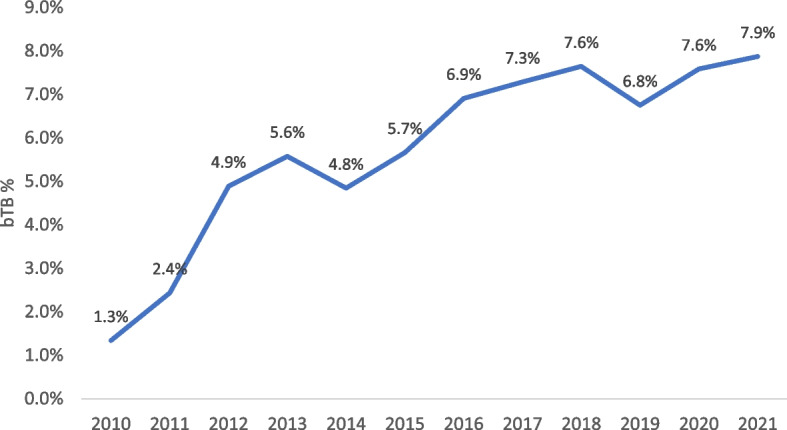
The percentage of bTB infected farms within the control zone (high prevalence area) each year

**Fig. 7:**
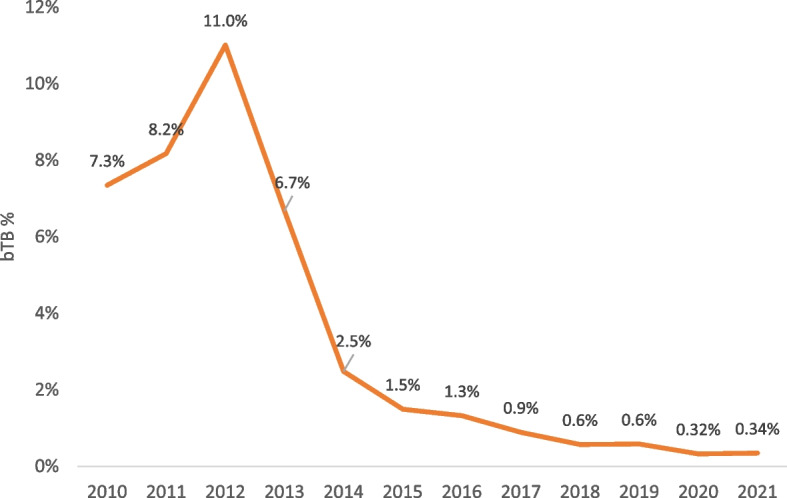
Percentage of bTB infected farms per year, eradication zone

Due to the limited coverage of bTB surveillance, the total number of surveillance farms that were considered infected, expressed as a percentage, was used to assess the annual dynamic changes in bTB rates. The results are shown in Figs. 6 and 7, where the percentage of infected farms as a proportion of the surveyed farms shows contrasting results between both zones in 2011 and 2021. In the control zone, which includes the high prevalence areas, there is evidence of an annual increase in the proportion of infected farms, reaching a high of 7.9% in 2021 (Fig. 6), while in the eradication zone the herd infection rate decreased to 0.3% over the corresponding period of time (Fig. 7).

In other studies, the vaccination field trial has shown that BCG could be a useful tool for reducing the bTB incidence in highly infected herds, where the rates in vaccinated and non-vaccinated heifers at the end of the trial were 0.76% and 2.27%, respectively [1]. This provides evidence to continue with the strategy to adopt vaccination in infected dairy herds within the official bTB program. It also helps to promote farmers’ interests in the apparent secondary benefits of vaccination, including positive indicators associated with improvements in milk production [8].

## Discussion

The annual reduction in bTB carcass condemnation rates is an important positive indicator of the program’s success, since it is a direct measure that impacts on the financial security and wellbeing of producers. As the program has progressed, visible lesions are now rarely seen in many herds. However, as a consequence of reduced infection levels, the economic losses due to carcass condemnation have also decreased for the farmers, which may serve to reduce incentives to continue and strengthen the bTB control programs, despite the continual presence of infection.

The progress of bTB control in the southern eradication zone in Chile has been similar to that experienced in the dairy production areas of Australia at the beginning of their brucellosis and tuberculosis eradication campaign (BTEC) [10]. In the eradication zone, the current strategy has been influenced by many factors including the previous bTB-free farm control programs in place, the dairy producers’ awareness of the bTB problem, stricter regulations and higher official budgets allocated for infected herds. In contrast, in the control zone, the number of bTB infected herds has increased and there is no evidence that the spread of infection has stopped.

The initial regulatory framework of the program tried to adopt the practicalities of implementation without interfering or challenging the livestock business and trade, while also recognising that the high bTB prevalence in dairy farms could result in herd depopulation at the producers’ expense. However, after ten years of the program, it is likely that the regulations may need to be updated, commensurate with the farmers’ commitment to disease control, and also to include beef breeders, since animal movements from this economically important sector may require stricter and additional rules.

The limited surveillance coverage in the control area and low level of herd classification are not unexpected given that regulations and incentives have not been sufficient to monitor herds, or indeed bTB herd classification in breeding systems or in dairy enterprises.

The commitment of the dairy industry, incentivized by the bonus paid per liter of milk to certified bTB free farms has not been sufficient to encourage dairy farms in the control area to maintain comprehensive disease control measures. Likewise, due to the fact that the largest proportion of infected farms is located in the area of breeding and fattening enterprises, it has become difficult to encourage their commitment to increased disease control measures, as it is a sector with varied interests that does not easily recognize bTB as its only problem.

## Conclusions

During the first 10 years of the bTB program in Chile, there has been progress in establishing rules and regulations to control the disease through defining protocols for the surveillance and classification of herds, as well as the use of the official traceability system as a tool to support the implementation of the program.

In the eradication zone, effective infection control has been achieved, mainly due to bTB-free farm certification, government support to clean up infected farms, as well as the beneficial interest of farmers exporting products or live animals. Despite the important progress achieved in the eradication zone, it will be a challenge to achieve eradication without compensation, not least because a much greater level of diagnostic coverage is required to identify all infected herds.

Within the control zone there is a contrasting epidemiological situation and no evidence of successful disease control. Although the dairy sector is aware of the impact of bTB, the high prevalence and the lack of compensation has driven the reluctance of producers to engage with the program. The farmers continue with a business-as-usual style of behavior. Given this lack of full commitment to the program, vaccination may provide an additional option to reduce bTB incidence. It may also provide a means to convince farmers of the benefits that may arise from increasing their commitment to disease control.

There is also a need to strengthen the agreement between the industry and the state, and to involve medium-sized farming enterprises who provide replacements for fattening. These enhanced agreements should address each of the following: a) reinforcing the implementation of bTB risk-based regulations, b) implementing movement controls to include beef breeders, both within and between areas; c) seeking joint industry-government schemes to financially compensate farmers for losses due to reactors (including removal of reactor animal to slaughter) or herd depopulation.

## Data Availability

Data sharing is not applicable to this article as no datasets were.generated or analyzed during the current study.

## Abbreviations

BCG: *Mycobacterium bovis* BCG vaccination
bTB: Bovine tuberculosis
BTEC: Brucellosis and tuberculosis eradication campaign
SAG: Livestock and Agriculture Service
SSA: Animal Health System
VL: Visible lesions
